# Endovascular embolization of visceral artery aneurysm: a retrospective study

**DOI:** 10.1038/s41598-023-33789-6

**Published:** 2023-04-28

**Authors:** Chi Gong, Ming-Sheng Sun, Rui Leng, Hua-Liang Ren, Kai Zheng, Sheng-Xing Wang, Ren-Ming Zhu, Chun-Min Li

**Affiliations:** 1 Department of Vascular Surgery, Beijing Chao-Yang Hospital, Capital Medical University, Beijing, China; 2 Department of General Surgery, Beijing Huai-Rou Hospital, Beijing, China; 3 Department of Vascular Surgery, Beijing Friendship Hospital, Capital Medical University, Beijing, China

**Keywords:** Outcomes research, Vascular diseases

## Abstract

To assess the safety and efficacy of endovascular embolization techniques, we compared the short- to medium-term prognosis of coil embolization for symptomatic visceral aneurysms (SVAA) and asymptomatic visceral aneurysms (ASVAA) to identify risk factors associated with 30-day mortality. Explore the symptom profile and intrinsic associations of SVAA. A retrospective study of 66 consecutive patients at two tertiary care hospitals from 2010 to 2020 compared the short- to mid-term outcomes of 22 symptomatic VAAs and 44 asymptomatic VAAs treated with coil embolization. Univariate and log-rank tests were used to analyze the prognostic impact of SVAA and ASVAA. SVAA group had significantly higher 30-day mortality than ASVAA group (2(9.1%) vs 0, P = 0.042), both patients who died had symptomatic pseudoaneurysms. Perioperative complications such as end-organ ischemia (P = 0.293) and reintervention (P = 1) were similar in both groups. No difference in event-free survival was identified between the two groups (P = 0.900), but we found that the majority of pseudoaneurysms were SVAA (4/5) and that they had a much higher event rate than true aneurysms. In addition, dyslipidemia may be an influential factor in the development of VAA (P = 0.010). Coil embolization is a safe and effective method of treatment for VAA. Most pseudoaneurysms have symptoms such as abdominal pain and bleeding, and in view of their risk, more attention should be paid to symptomatic patients and the nature of the aneurysm should be determined as soon as possible to determine the next stage of treatment.

## Introduction

Visceral artery aneurysm (VAA) is a rare condition with a worldwide prevalence of approximately 1% but its rupture is associated with a high risk of a lethal outcome^1^. However, autopsy reports suggest that VAAs may be more common than abdominal aortic aneurysms^2^. With changing demographics and advances in imaging technology, an increasing number of cases involving VAA are anticipated in future^3^. VAA can be divided into true aneurysms and pseudoaneurysms. True aneurysms have an intact vascular structure and are caused by the expansion of the entire blood vessel^4^. Pseudoaneurysms are caused by rupture of the intima and media and then swelling of the adventitia, usually due to infection, iatrogenic injury, and inflammatory reaction; these are very prone to rupture. In literature pseudoaneurysms tend to be more symptomatic than true aneurysms^2,5^. The most common VAA is splenic aneurysm with an incidence of approximately 60%, followed by hepatic aneurysm which accounts for approximately 20% of cases^6,7^.

Most VAAs are usually asymptomatic and found incidentally on routine abdominal imaging and most of those that do have symptoms involve abdominal pain, with a small number of patients experiencing ischemic or hemorrhagic symptoms. Intra-abdominal hemorrhage due to VAA perforation has a mortality rate of 20–100%^8^. Therefore, surgical treatment to prevent VAA expansion and rupture is a common measure in clinical practice. In recent years, with the rapid development of endovascular therapy techniques, coil embolization has become the most commonly used procedure for the treatment of VAA^9^. Moreover, endovascular treatment has been proven to be a safe and effective treatment with fewer complications and shorter hospitalization periods than open surgery^10,11^.

Few studies have investigated the embolization treatment of symptomatic VAA (SVAA) and asymptomatic VAA (ASVAA). Therefore, the purpose of our current study was to compare the perioperative and follow-up outcomes of SVAA and ASVAA after coil embolization.

## Materials and methods

This was a retrospective double-center study that was approved by Beijing Chaoyang hospital ethic committee and Beijing Friendship hospital ethic committee. A statement that all methods in this study were performed in accordance with relevant guidelines and regulations. We reviewed the databases of Beijing Chaoyang Hospital, Capital Medical University, and Beijing Friendship Hospital, Capital Medical University, between 2010 and 2020. Written informed consent was obtained from every patient and from legal guardians of the dead participants.

### Patients

Usually we confirm the diagnosis of VAA by angio-CT and after that, if the patient's VAA needs to be intervened by endovascular techniques, both diagnostic angiography and endovascular treatment are performed.

**Figure 1:**
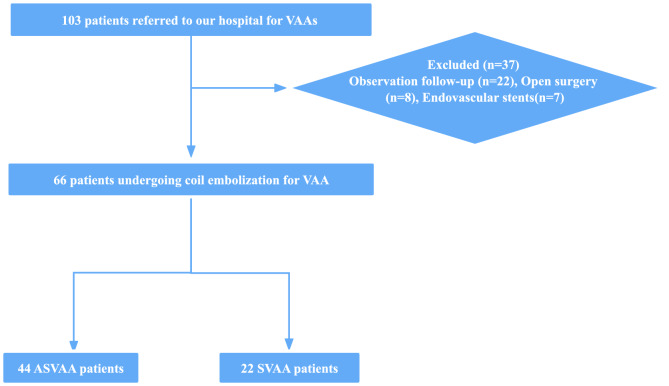
Patient flow chart. *VAA* visceral artery aneurysm, *SVAA* symptomatic visceral artery aneurysm, *ASVAA* asymptomatic visceral artery aneurysm.

The treatment modalities for all 103 VAA patients included conservative treatment with regular observation (22/103), open surgical treatment (8/103), stenting (7/103), and coil embolization (66/103). We selected 66 patients who underwent coil embolization from 103 patients diagnosed with VAA and classified these as SVAA and ASVAA, including 57 cases of coil-filled aneurysms and 9 cases of embolization of the aneurysm-carrying artery. The screening process is shown in Fig. 1.

### Coil embolization and management

At present, the surgical indications of VAA are mainly based on the size, nature and location of the aneurysm, as well as the patient's gender, fertility potential, life expectancy, and physical condition. There is no absolute contraindication to coil embolization therapy. Patients with hemodynamic instability after resuscitation, contrast allergy, ruptured aneurysms, patients with pseudoaneurysms who have undergone embolization, and infections in the target vessel lumen are relative contraindications to this procedure.

All 66 patients were treated with coil embolization via the femoral route by experienced vascular surgeons. After introduced the 6F sheath, followed by using the 5-6F catheter to the parent vessel that allows access to the aneurysm. Filling of true aneurysms with coils and preserving the parent artery is the key point in the procedure. For patients with pseudoaneurysm or rupture aneurysm we perform “sandwich technique” or stent-assisted coiling. The sandwich technique, which involves embolization of the inflow and outflow vessels of the aneurysm to completely exclude the aneurysm from circulation, needs to be explained. This technique prevents side-branch blood flow back into the aneurysm. Due to anatomic limitations or aneurysm complexity, stent is not always possible. Detachable coils can provide predictable and controlled deployment. The aneurysms were packed as densely as possible with detachable coils, including Boston Scientific’s interlocking detachable coils and Cook's detachable coils.

Technical success was defined as complete aneurysm exclusion at ultimate angiographic control. Clinical success refers to 30-day clinical outcomes with clinical and imaging data according to established guidelines^12^. According to the Interventional Radiology Standards Board Guidelines, complications included puncture site complications, aneurysm rupture, end-organ ischemia, deep vein thrombosis, heart attack, and stroke^13^.

Follow-up examination using conventional or magnetic resonance angiography was performed 3–6 months after the initial treatment to assess the efficacy of coil embolization and the aneurysm status. We used color Doppler ultrasound or enhanced computed tomography (CT) to assess complications and distal organ ischemia when necessary. A member of the study team was responsible for collecting clinical information, including patient demographics, treatment details and impact, complications, and follow-up results; this person was not involved in the treatment process for any patients. Aneurysms volume measured by imaging the specific numerical calculation, the whole calculation process on a website (https://www.angiocalc.com/).

Demographic characteristics collected for this study included age, gender, and co-morbidities (hypertension, diabetes, smoking, dyslipidemia, coronary artery disease, chronic obstructive pulmonary disease, renal dysfunction, and cirrhosis). Treatment details included the main symptoms, treatment strategies, aneurysm characteristics (size, location, pseudoaneurysm, anatomical variants, and rupture status), and reinterventions.

### Endpoints

The primary endpoint for this study was overall mortality. The secondary endpoints were perioperative complications and reinterventions.

### Statistical analysis

Data were analyzed by using SPSS Statistics software (version 25.0 for Windows, SPSS, Chicago, Illinois). Continuous variables were analyzed using a two-tailed Student t test and the significance of relationships between variables was tested by the Chi-squared test or Fisher’s exact test coefficient of rank correlation. The endpoint event times were compared between groups using Kaplan–Meier plots. A significance level of α = 0.05 was defined.

## Results

### Characteristics of patients and aneurysms

The 66 patients included in the study were divided into two groups as symptomatic (group A) and asymptomatic (group B); the demographic characteristics are shown in Table 1. No significance difference was found between the two groups in terms of age (A vs B, 56 ± 10.64 vs 59 ± 10.23; P = 0.422). There were more female patients than male patients in both groups, but there was no significant difference in gender distribution between the two groups. One patient in group A had cirrhosis, although there was no significant difference between the two groups in this respect (P = 1). The proportion of patients with dyslipidemia was greater in group A than in group B, of significance [12(54.5%) vs 10(22.7%), P = 0.010].

**Table 1:** Demographic characteristics of symptomatic visceral artery aneurysm (SVAA) and asymptomatic visceral artery aneurysm (ASVAA).

	SVAAs	ASVAAs	P value
Age ± SD (years)	56 ± 10.6	59 ± 10.2	0.422
Sex, female	12 (54.5)	27 (61.4)	0.595
Co-morbidities			
Hypertension	13 (59.1)	21 (47.7)	0.384
Diabetes	5 (22.7)	5 (11.4)	0.225
Smoker	6 (27.3)	15 (34.1)	0.575
Dyslipidemia	12 (54.5)	10 (22.7)	0.010
Cardiovascular disease	6 (27.3)	7 (15.9)	0.274
COPD	0	2 (4.5)	0.549
Renal dysfunction	1 (4.5)	1 (2.3)	1
Liver cirrhosis	0	1 (2.3)	1
Pregnancy	0	0	

*COPD* chronic obstructive pulmonary disease.

**Figure 2:**
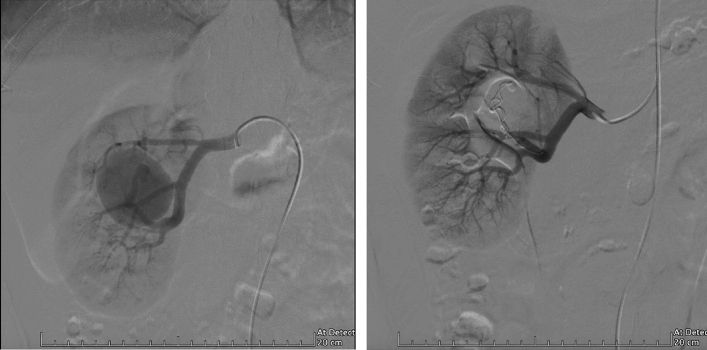
(**A**) Renal artery aneurysm (RAA) arising from a branch of the renal artery. (**B**) Post coil embolization angiogram showing complete exclusion of aneurysm.

**Figure 3:**
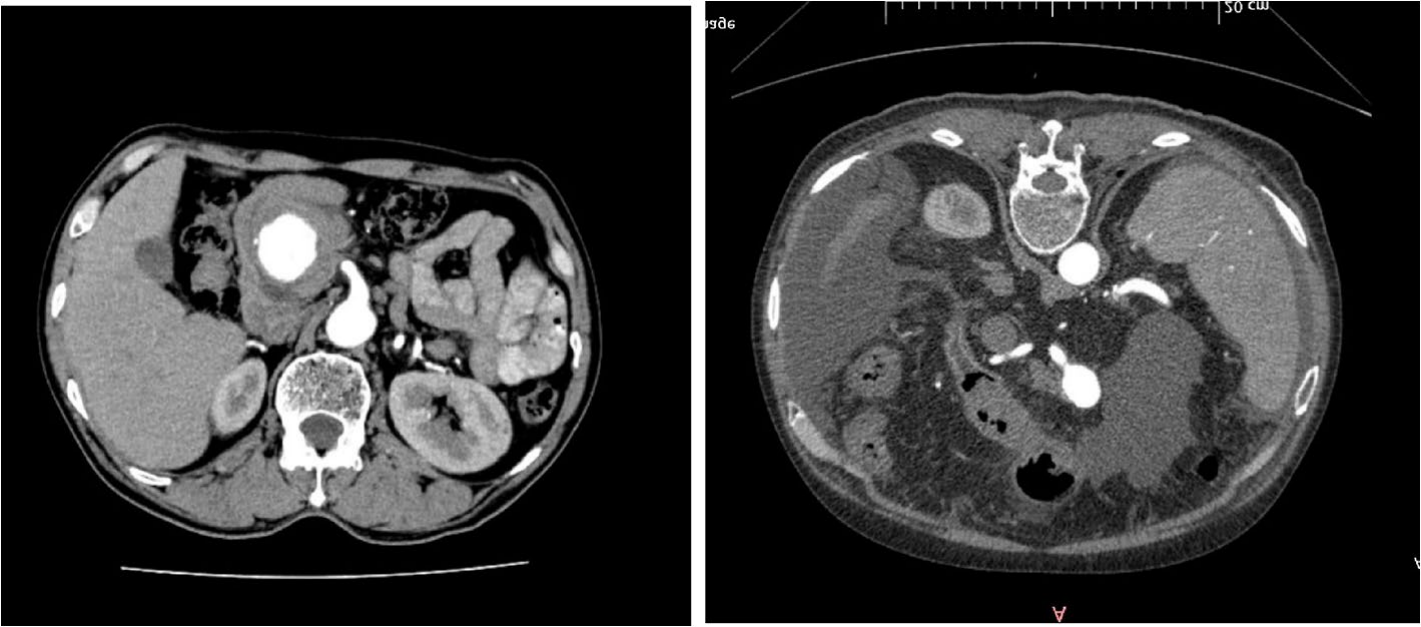
(**A**) CT imaging of pseudoaneurysm. (**B**) CT imaging of true aneurysm.

The aneurysm characteristics of the two groups of patients are shown in Table 2. The median aneurysm volume in group A was smaller than that in group B, although this was not significant [1591 mm^3^ (629, 5269) *vs* 2498 mm^3^ (1414, 5095), P = 0.174]. Splenic aneurysms accounted for the majority of all aneurysm types in both Group A and Group B. In group A, the proportion of patients with SAA (P = 0.005) was significantly higher than in group B, while the proportion of patients with RAA (P = 0.047) was significantly lower than in group B (Fig. 2). Patients with pseudoaneurysms were almost exclusively in group A, with a significantly higher proportion in group A than in group B [(4(18.2%) *vs* 1(2.3%), P = 0.039] (Fig. 3). In our study, there were three ruptured aneurysms, two of which were in group A. Both were male patients with pseudoaneurysm. In the first patient, who underwent radical cholangiocarcinoma surgery for bile duct cancer, sudden vomiting of approximately 200 ml of blood occurred on the 19th postoperative day; this was accompanied by nausea and vomiting, and signs of hemorrhage such as increased heart rate and decreased blood pressure. Hemorrhage from a pseudoaneurysm of the hepatic artery was detected during interventional angiography, and bleeding continued after embolization treatment. Hepatic artery gastroduodenal artery ligation was performed to stop the bleeding, and a postoperative intestinal fistula developed accompanied by a progressive reduction in hemoglobin. He eventually died after resuscitation. Another patient died due to rupture of a splenic pseudoaneurysm; he had just started interventional embolization, but hemoglobin decreased progressively. Therefore, open exploration was performed to clear the hemorrhage, the postoperative heart rate decreased, and he died after resuscitation. Rupture of a pseudoaneurysm occurred in a female patient in group B; after examination, we found two true aneurysms in the middle segment of the splenic artery. This patient was asymptomatic at first so we performed endovascular embolization treatment. After the operation, the patient showed signs of bleeding, including a progressive decrease in hemoglobin with fever. We performed an open exploration and found that the aneurysm had ruptured. The patient recovered well after removal of the spleen and was discharged successfully.

**Table 2:** Aneurysm characteristics between symptomatic visceral artery aneurysm (SVAA) and asymptomatic visceral artery aneurysm (ASVAA) groups.

	SVAAs	ASVAAs	P-value
Diameter (mm)	28 (16, 36)	21 (16, 25)	0.119
Aneurysms volume (mm^3^)	1591 (629, 5269)	2498 (1414, 5095)	0.174
Location			
SAA	10 (45.5)	35 (79.5)	0.005
HAA	3 (13.6)	1 (2.3)	0.104
RAA	9 (40.9)	8 (18.2)	0.047
Pseudoaneurysms	4 (18.2)	1 (2.3)	0.039
Ruptured aneurysms	2 (4.5)	1 (2.3)	0.256

*SAA* splenic artery aneurysm, *HAA* hepatic artery aneurysm, *RAA* renal artery aneurysm.

### Complications and follow-up

Patient-specific complications and adverse event follow-up results are shown in Table 3. We found no patients with arterial dissection or thrombosis after surgery. In one patient in group B there was flow recirculation of the aneurysm after embolization. There was no significant difference in the length of stay when compared between the two groups. There were six cases of surgery-related complications: the one case (4.5%) of end-organ ischemia in the A group mentioned above and five cases (11.4%) of end-organ ischemia and recirculation in B group (P = 0.364). End-organ ischemia was the most common complication in patients after coil embolization, also in groups A and group B [1 (4.5%) *vs* 4 (9.1%), P = 0.293], respectively. The reintervention rate was similar in both groups [2 (9.1%) *vs* 3 (6.8%), P = 1]. In group A, reintervention was performed due to pseudoaneurysm rupture, while in group B, reoperation was performed because of aneurysm rupture, recanalization, and hemorrhage. The two patients who died within 30 days were both in group A; the 30-day mortality rate was significantly higher in group A than in group B [2(9.1%) *vs* 0, P = 0.042]. Both patients who died were male and both had pseudoaneurysms.

**Table 3:** Complications and perioperative outcome between symptomatic visceral artery aneurysm (SVAA) and asymptomatic visceral artery aneurysm (ASVAA) groups.

	SVAA (n = 22)	ASVAA (n = 44)	P value
Length of stay (days)	11.5 ± 8.8	8.98 ± 2.8	0.227
Complication	1	5	0.364
End-organ ischemia	1 (4.5)	4 (9.1)	0.293
Arterial dissection and thrombosis	0	0	
Recirculation	0	1 (2.3)	0.476
Reintervention	2 (9.1)	3 (6.8)	1
Thirty-day mortality	2 (9.1)	0	0.042

**Figure 4:**
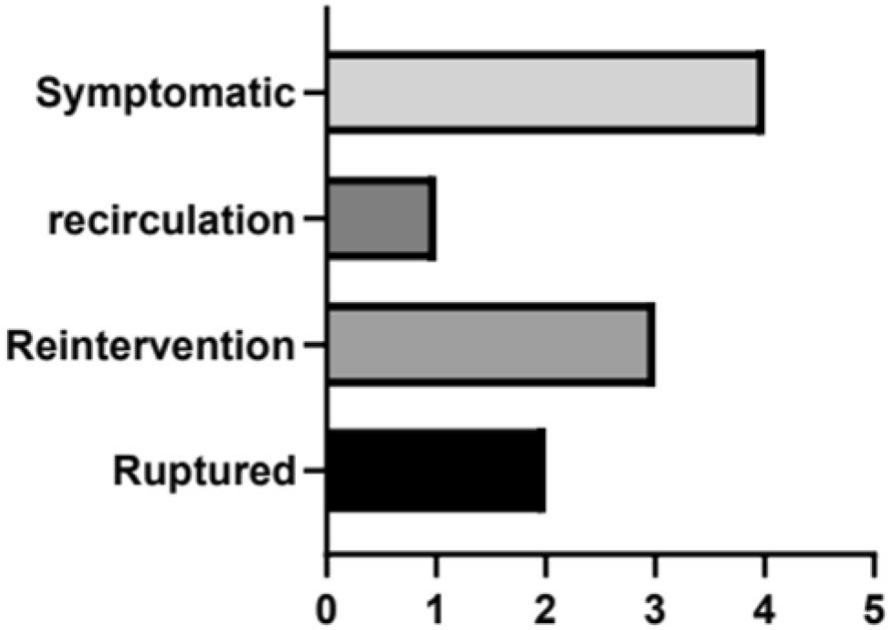
Events related to pseudoaneurysm.

As a matter of note, pseudoaneurysms were associated with a considerable number of complications, as shown in Fig. 4. In total, 80% of patients with pseudoaneurysms were symptomatic, and the two patients who died from ruptured pseudoaneurysms were part of this group. One patient with a pseudoaneurysm showed little blood flow initially, and subsequent imaging findings showed that the aneurysm had no blood flow. In addition to the two patients who died from rupture and underwent urgent secondary intervention after rupture, one patient experienced a progressive postoperative reduction in hemoglobin; we re-embolized his hepatic pseudoaneurysm and fortunately he was saved.

**Figure 5:**
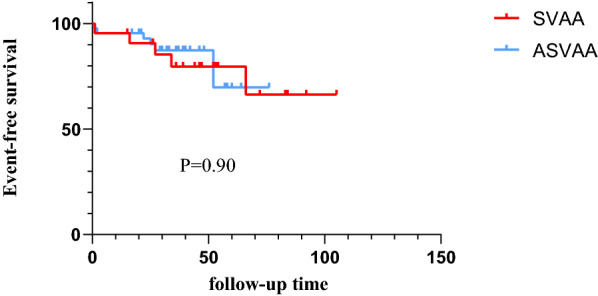
Kaplan–Meier analysis for event-free survival for symptomatic visceral artery aneurysm (SVAA) and asymptomatic visceral artery aneurysm (ASVAA) during follow-up.

The follow-up period of this study was approximately 40.56 ± 21.36 months (range 1–105 months). All patients underwent re-imaging and one patient received recanalization. Two deaths occurred during the follow-up period, one from a ruptured pseudo-splenic aneurysm and one from a ruptured hepatic artery after embolization. No statistic significant difference was detected in terms of event-free survival (Fig. 5) between the A group and B group (77.3% *vs* 84.1%, P = 0.900).

## Discussion

Coil embolization is widely used in the treatment of VAA because of its minimally invasive nature, short recovery time, and low rates of morbidity and mortality^14^. Most VAAs are asymptomatic^15^, and 36.9% (38/103) of all VAA patients in this study had acute symptoms due to rupture and gastrointestinal bleeding. Symptomatic aneurysms are an indication for treatment, although few studies have compared SVAA and ASVAA.

There are currently several options available for the endovascular treatment of VAA, including embolization, covered stents and bypass stents, according to a recent report by Venturini^15^. Among the different materials that can be used for embolization techniques are coils, glue, non-adhesive embolizing agents, plugs and particles. The coil embolization used in this study is the most common endovascular technique used to treat VAA. Appropriately sized coils, typically exceeding 20% of the vessel lumen, should be selected. Coils that are too large cannot be adequately expanded, weakening their thrombogenic effect, and coils that are too small may increase the risk of re-circulation and distal displacement. Although endovascular techniques have the advantage of less invasive and easier handling, there are still some technical challenges in the endovascular treatment of giant VAAs (generally defined as VAAs over 5 cm in diameter). In the Tipaldi’s study, endovascular treatment of giant VAA achieved satisfactory timely and long-term results, proving to be an effective and minimally invasive solution^16^. There were two cases of giant VAAs in this study, treated by sandwich technique embolisation, one of which was a male patient with a splenic pseudoaneurysm who later died due to a ruptured aneurysm. In addition, Cappucci's study showed that endovascular stenting for VAA is safe and effective in the long term, and that the technique has a high success rate with a low complication rate^17^. However, endovascular stenting is not foolproof and can lead to a number of complications. For example, Tipaldi reported extravascular migration of stent, a rare and easily overlooked complication. They collected five patients with splenic artery aneurysms involving stent extravascular migration, and indicates its potential to cause serious complications such as haemorrhage^18^.

For VAAs that meet the indications, whether they are true aneurysms or pseudoaneurysms, intervention is recommended by guidelines as a matter of urgency due to their susceptibility to rupture. Mortality after VAA rupture is acknowledged to be high; the overall 30-day mortality rate in our study was approximately 3%. All deaths occurred in the A group; the mortality rate was significantly higher than the B group (P = 0.042); this result was similar to that published previously^6,19^. The event-free survival rate was higher in the B group (84.1%) than in the A group (74.3%), although there was no significant difference between the two groups (P = 0.900). Importantly, the two patients who died in this study were also the patients who died in the A group; both had pseudoaneurysms, except that one was from the splenic artery and the other was from the hepatic artery. Considering the high mortality rate following the rupture of pseudoaneurysms, coil embolization is performed immediately after a confirmed diagnosis. In a previous study, Carr et al. demonstrated a 50% mortality rate (3/6) after the successful emergency embolization of pseudoaneurysms^20^; this is similar to the 40% mortality rate for pseudoaneurysms observed in the current study. Consequently, pseudoaneurysms still have a high mortality rate even when successfully treated with embolization. Based on our data, 60% (3/5) of patients with pseudoaneurysms require reintervention; this may be related to their specific and fragile structure.

Pseudoaneurysms are mainly caused by inflammation and trauma, with rupture of the intima and the resulting episcleral bulge, owing to the thinner abnormal vascular structures. Tétreau's study showed no significant difference in the mean diameter of intact and ruptured pseudoaneurysms, and therefore pseudoaneurysms should be treated irrespective of size^21^. Smaller pseudoaneurysms tend to be detected only when they show dangerous symptoms such as bleeding, while larger pseudoaneurysms can be found incidentally on imaging. The incidence of pseudoaneurysm rupture can be as high as 80%, depending on the location, and the mortality rate can be as high as 100% if left untreated^22,23^. In the last century, immediate open surgery was recommended for pseudoaneurysms due to war or traumatic causes that made them difficult to treat or impossible to heal. With the rapid development of endovascular techniques over the last two decades, and the fact that endovascular treatment has been proven to be safe and effective in most studies, endovascular treatment has become the first choice of more and more vascular surgeons for the treatment of pseudoaneurysms^24,25^. Theoretically, the use of coils or hydrogels to occlude a pseudoaneurysm, followed by an endoluminal stent to maintain revascularization, is a safer and more reliable approach, and also frequently used in the treatment of intracranial aneurysms. One reason for this is that in some wider aneurysms of the neck, simple tamponade has the potential to allow the coil or tamponade to move into the vessel postoperatively, inadvertently increasing the risk of thrombosis or embolism. Direct embolization of vessels in focal sources such as the duodenal arteries appears to be a good approach, but some vessels are not suitable for such treatment, the anatomy of patients is diverse and complex, and many visceral arteries are too small or too tortuous to allow for successful stent placement, thus resulting in a limited selection of stent grafts.

Approximately 62% of aneurysms are reported to be caused by atherosclerosis^26^; lipoproteins are known to play an important role in atherosclerosis. The proportion of patients with dyslipidemia was higher in A group than in the B group (P = 0.010). Most studies have concluded that total cholesterol and low-density lipoprotein levels are positively associated with the risk of aneurysm. For example, Kubota et al. investigated 13,683 participants without a history of aneurysm surgery over a 2-year period; restricted cubic spline analysis demonstrated a positive dose–response relationship between plasma lipoprotein (a) with abdominal aortic aneurysm, with a steep increase in the risk of AAA above the 75th percentile^27^. In another study, Huang et al. concluded that high-density lipoprotein (HDL) was negatively associated with the risk of growth and rupture of intracranial aneurysms (P = 0.002)^28^. In contrast, however, other studies have shown that overly low triglyceride and low-density lipoprotein (LDL) levels significantly increase the risk of vascular rupture and bleeding. Rist et al. showed that LDL-C levels below 70 mg/dL and low triglyceride levels significantly increased the risk of hemorrhagic stroke; these authors suggested that low cholesterol leads to necrosis of the smooth muscle cells in the middle layer of the artery and that endothelial damage is more likely to lead to microaneurysms, which are often found in patients with cerebral hemorrhage^29^. We next plan to further specify the lipid data of VAA patients to analyze the effect of various lipoproteins on the prognosis of VAA.

In general, coil embolization therapy has a clear advantage for the parenchymal organs (e.g., splenic aneurysms, hepatic aneurysms), where open surgical treatment may sacrifice the spleen or part of the liver. Splenic infarction accounts for the majority of end organ ischemia and has the potential to occur when the embolization of more distal splenic artery lesions is performed. In a retrospective study of 253 visceral aneurysms, 20 SAA patients were treated with splenic artery embolization^6^. Two cases of splenic infarction and one case of pancreatic abscess were found in this group; all of these aneurysm occurrences were found in the splenic bed.

The mortality rate within 30 days after surgery in our study was approximately 3%, which is much lower than that in other studies^30,31^. However, our study did not include all types of visceral aneurysms, such as mesenteric aneurysms, pancreaticoduodenal aneurysms, and gastroduodenal aneurysms. In addition, due to the low prevalence of VAA, the number of cases that could be included in this study was limited.

## Conclusions

Coil embolization is a safe and effective method of treatment for VAA. Lipid levels have the potential to influence the characteristics and progression of aneurysms. We observed no significant difference in event-free survival at midterm follow-up between cases of SVAA and ASVAA. However, most pseudoaneurysms have symptoms such as abdominal pain and bleeding, and in view of their risk, more attention should be paid to symptomatic patients and the nature of the aneurysm should be determined as soon as possible to determine the next stage of treatment. Furthermore, asymptomatic pseudoaneurysms as well as symptomatic true aneurysms, should be carefully assessed and could be treated, according to international guidelines, due to the increased risk of rupture^32^.

## Data Availability

The datasets used and/or analysed during the current study available from the corresponding author on reasonable request.

## Abbreviations

VAA: Visceral artery aneurysm
SVAA: Symptomatic VAA
ASVAA: Asymptomatic VAA
SAA: Splenic artery aneurysm
HAA: Hepatic artery aneurysm
RAA: Renal artery aneurysm
